# Enhancing Dermal Absorption of Cosmeceuticals: Innovations and Techniques for Targeted Skin Delivery

**DOI:** 10.1111/jocd.70514

**Published:** 2025-10-23

**Authors:** Yichao Peng, Lixia Yue, Yuanhua Cong

**Affiliations:** ^1^ School of Pharmacy Shanghai University of Traditional Chinese Medicine Shanghai China; ^2^ State Key Laboratory of Systems Medicine for Cancer, Shanghai Cancer Institute, Renji Hospital, School of Medicine Shanghai Jiao Tong University Shanghai China; ^3^ School of Medical Instrument and Food Engineering University of Shanghai for Science and Technology Shanghai China; ^4^ Institute of Advanced Technology University of Science and Technology of China Anhui China

**Keywords:** cosmetics, invasive, non‐invasive, skin barrier

## Abstract

**Background:**

As beauty consumption upgrades and skincare concepts evolve toward science‐backed efficacy, the field of cosmetology has put forward higher requirements for the dermal absorption efficiency of efficacious products. Notably, amid the global medical aesthetics market's 12.8% Compound Annual Growth Rate (projected to reach $35,327.5 million by 2030), rising demand for high‐efficacy cosmeceuticals has amplified the significance of a longstanding issue: the low transdermal efficiency of active ingredients due to skin barrier and molecular constraints. Various studies have explored different dermal absorption promotion methods, but there is a lack of a systematic review that encompasses all types of permeation promotion technologies.

**Aims:**

This review aims to systematically sort out the mechanism of dermal absorption and comprehensively summarize the commonly used permeation methods, in order to provide technical solutions for breaking through the barriers of active ingredient delivery and realizing the targeting effect of the skin in the deep layers of the skin.

**Methods:**

We conducted a comprehensive review and analysis of existing literature related to dermal absorption promotion methods, systematically organizing the mechanisms of dermal absorption and integrating the commonly used permeation approaches.

**Results:**

Active ingredients show low transdermal efficiency due to skin barrier and molecular constraints, with permeation via epidermal pathways and appendages. Non‐invasive (chemical enhancers, nanocarriers, ultrasound) and invasive (microneedles, electroporation, needle‐free injectors) techniques exist, with combinations boosting efficacy.

**Conclusions:**

Appropriate dermal technology is crucial to solve the problem of low dermal efficiency of active ingredients. The systematic sorting of dermal absorption mechanisms and the comprehensive summary of commonly used permeation methods in this review can provide effective technical support for the development of high‐efficacy cosmeceuticals and the improvement of dermal penetration efficiency in the field of cosmetology.

## 1 Introduction

The pursuit of beauty and health remains a persistent core demand among female consumers. Particularly against the backdrop of sustained economic growth, more and more resources are being invested in skin care and other cosmetic products. As consumers have more resources at their disposal, they are demanding higher skincare results, and the demand for minimally invasive and non‐invasive cosmetic procedures is growing. In recent years, the global cosmetic dermatology market size has continued to grow, and the global medical aesthetics market is expected to reach $35,327.5 million by 2030, growing at a Compound Annual Growth Rate of 12.8% from 2024 to 2030 [1].

From a formulation design perspective, the efficacy of skincare products is closely linked to three factors: the logic of the formulation design, the selection of active ingredients, and the use of appropriate methods to enhance their bioavailability. In contrast to topical pharmaceuticals, skincare products are products that consumers use every day for a long period of time, and the process of enhancing dermal penetration may alter the barrier structure of the skin [2, 3], so the requirements for safety are extremely stringent, and a comprehensive review of dermal penetration methods is necessary. For the development of the cosmetic industry, this is also the key and necessary path for technological progress.

As the largest organ of the human body, the skin not only has the functions of protection, temperature regulation, and sensation [4], but is also an important target organ for cosmetic skin care. With the increasing pursuit of skin health and beauty, how to safely and efficiently deliver the active ingredients to the target areas of the skin has become a key issue in the field of skin aesthetics. As a non‐oral active ingredient delivery method, dermal absorption technology can deliver active ingredients and other ingredients directly to the inner skin [5], showing great potential for application in the field of dermatological aesthetics.

This review systematically clarifies the permeability barrier of the skin, and then summarizes and describes common techniques for enhancing dermal absorption.

## 2 The Structure of the Skin

As the body's first line of defense, the skin not only prevents the loss of internal moisture to a certain extent, but also has the ability to absorb and permeate external substances [6]. The skin can be divided into three layers: the epidermis, dermis, and subcutaneous tissue [7]. The epidermis is the outermost layer of the skin, and it is primarily composed of keratinocytes. Based on the degree of cellular keratinization, it can be divided into the basal layer, spinous layer, granular layer, clear layer, and cornified layer [8]. Proliferating keratinocytes migrate from the basal layer to the skin surface, where they differentiate into cornified cell [9]. The cornified layer is the outermost layer of the epidermis [10], consisting of cornified cells embedded in a lipid bilayer matrix, which can be likened to a “brick wall structure” [11] with the cornified cells as the “bricks” and the lipid intercellular matrix as the “mortar” [12]. The dermis is made up of fibrous collagen and elastic tissue, and it contains blood vessels, nerves, and sensory receptors. It acts as a hydrophilic layer that supports and nourishes the epidermis while connecting it with the subcutaneous tissue [9, 13]. The subcutaneous tissue, found deep within the dermis, is primarily composed of adipose tissue in most cases [14]. It contains abundant capillaries that allow active absorption through the skin to circulate throughout the body. Additionally, the thickness and condition of the epidermis are also influenced by factors such as race, gender, and anatomical location [15].

## 3 Pathways of Skin Permeation

**FIGURE 1:**
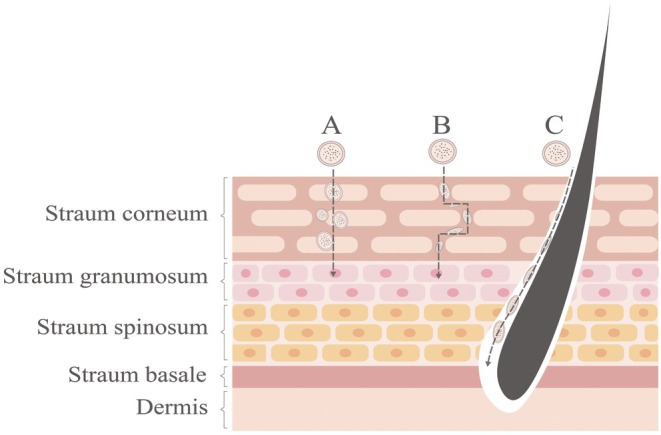
Pathways of skin permeation. (A) the intercellular pathway. (B) transcellular pathway. (C) the skin appendage pathway.

The pathways of skin permeation can be divided into two main routes: the intact epidermal pathway and the skin appendage pathway [16], as illustrated in Figure 1. The intact epidermal pathway is the primary route of transdermal penetration, where the stratum corneum, consisting of corneocytes and their intercellular spaces, exhibits lipid‐like properties that facilitate the permeation of lipophilic, non‐ionized actives [17]. Within the intact epidermal pathway, permeation can be further subdivided into two pathways: the intercellular pathway, which occurs through the intercellular gaps, and the transcellular pathway, which involves transport across the corneocytes [18]. Through the transcellular pathway, hydrophilic or polar solutes can be transported intracellularly, whereas the intercellular gaps facilitate the diffusion of lipophilic or non‐polar solutes through the continuous lipid matrix [19]. The skin appendage pathway involves the sebaceous glands, hair follicles, and sweat glands [20, 21]. They account for only about 0.1% of the total surface area [22], they serve as minor routes of skin absorption, particularly for large molecules and ionized actives [23].

## 4 Techniques for Dermal Absorption

Poor skin permeability is one of the major challenges facing transdermal active ingredient delivery systems [3]. Notably, this challenge is partly governed by the “500 Dalton rule”, which posits that compounds with a molecular weight exceeding 500 Da struggle to penetrate the stratum corneum of normal skin, whereas those below this threshold permeate more readily through epidermal pathways [24]. This presents a particular hurdle for larger active ingredients such as peptides and proteins, which cannot penetrate the skin autonomously. In order to overcome the low permeability of the stratum corneum, a natural barrier, and to improve the efficiency of dermal absorption of the active ingredient, it is particularly important to select a reasonable transdermal technology. The following summarized permeation‐promoting techniques are based on the improvement of the stratum corneum barrier, with invasive and non‐invasive modalities listed. Non‐invasive approaches mainly aim to enhance the permeability of corneocytes or modify the permeability of the intercellular lipid matrix to facilitate absorption. Invasive methods involve disrupting the stratum corneum to improve penetration into the deeper layers of the skin.

### 4.1 Non‐Invasive Techniques

#### 4.1.1 Chemical Permeation Enhancers

Chemical permeation enhancers (CPE) refer to a class of chemical substances that can accelerate the penetration of actives into the skin. They can increase active permeation by mechanisms such as enhancing lipid fluidity, altering keratin to increase its hydration, increasing the thermodynamic activity of actives, and improving active solubility, which are related to their own chemical properties [25, 26, 27]. Common CPE include alcohols, alkyl ethers, fatty acid derivatives, amides, sulfoxides, pyrrolidones, azepanones, terpenes, hyaluronic acid derivatives, various surfactants, sodium dodecyl sulfate, etc. [3, 26, 28]. Zhao et al. [29] found that the combination of azones and salicylic acid significantly enhanced the permeability of salicylic acid at lower concentrations, maximizing its anti‐aging effect.

Although CPE can enhance active permeation and have low costs, they can be irritating and toxic to the skin when the concentration of the enhancer is too high or when the contact time with the skin is too long [3, 30]. For example, the addition of surfactants may cause allergic contact dermatitis [31]. Currently, high‐throughput screening techniques can be used to screen suitable permeation enhancer [32], and the combination of multiple permeation enhancers and the use of biodegradable enhancers can enhance permeation, reduce toxicity, and irritation [25]. For example, when N‐lauroyl‐L‐arginine and dehydrated mannitol mono‐laurate (S20) are mixed, the permeation enhancement is increased while the irritation is reduced [33]. Biodegradable permeation enhancers can degrade into non‐toxic compounds when they interact with the viable skin layer, such as amino acid‐based amphiphiles [25].

### 4.2 Nanotechnology

**FIGURE 2:**
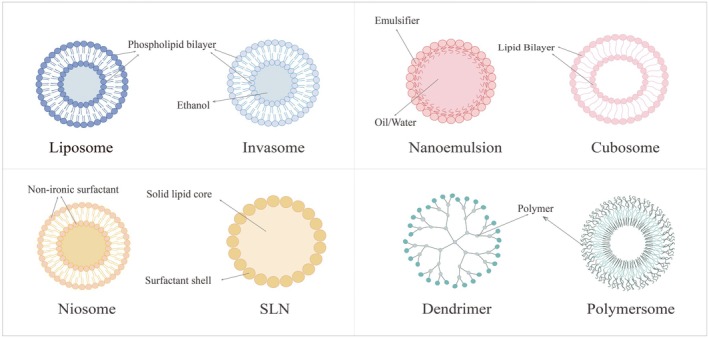
Diagrammatic structure of nanocarriers.

According to the definition in the field of materials science, the particle size of a nanocarrier is smaller than 100 nm [34, 35]. However, compared to traditional active delivery methods, nanocarriers provide a passive active delivery strategy that is considered safer and faster [36, 37]. In the past few decades, there have been many innovative studies based on nanotechnology. These studies have significantly improved the transdermal effects of active ingredients and reduced side effects of materials by changing actives and other components at the nanoscale using nanotechnology methods [38]. Nanocarriers have also been widely used in various pharmaceutical fields, and they are commonly used in cosmetic products now [34, 39], such as anti‐aging care, cosmetics, sunscreens, and hair care products [39]. Nanocarriers not only enhance the efficacy of cosmeceutical products, providing better and longer lasting effects, but also help improve the aesthetic appeal of the product [40]. Common nanocarriers include liposomes [41], invasomes [42], nanoemulsions [43, 44], cubosomes [45, 46], dendrimers [47], solid lipid nanoparticles [48, 49], polymersomes [50, 51], and niosomes [52, 53], as illustrated in Figure 2.

#### 4.2.1 Liposomes

Liposomes are colloidal spheres with a dual lipid bilayer structure that is self‐assembled in a solution from amphiphilic phospholipid molecules. They can be made from cholesterol and naturally non‐toxic phospholipids [54]. Due to their unique dual lipid bilayer structure, liposomes are used as carriers for both lipophilic and hydrophilic active components [55]. Hydrophilic substances are encapsulated in the internal aqueous compartment, while lipophilic actives are mainly enclosed within the lipid bilayer [56]. Liposomes not only have good biodegradability, biocompatibility, non‐toxicity, and non‐immunogenicity [57], but also improve the stability of and bioavailability of actives [56, 58]. Liposomes mainly promote dermal active absorption by disrupting the lipid arrangement of the stratum corneum, thereby changing the fluidity or flexibility of the stratum corneum lipids and increasing its permeability [59]. Coenzyme Q10 (CoQ10) is commonly used as an anti‐aging and anti‐wrinkle agent [60]. Lee et al. [61] encapsulated CoQ10 in liposomes composed of soy phosphatidylcholine and alpha‐tocopherol, resulting in a 2‐fold increase in its accumulation in the skin compared to non‐liposomal formulations. In the experiment, 0.1 mg/mL free‐form and liposome‐encapsulated CoQ10 were applied to rat skin, and after 4 h, the concentration of the free‐form in the skin was below 2 μg/g, while the concentration of the liposome‐encapsulated form was close to 4 μg/g. However, traditional liposomes have poor stability and transdermal effects, and a low encapsulation rate for some water‐soluble actives. To overcome this disadvantage, we have developed new types of liposomes based on traditional liposomes by changing the formulation. For example, the addition of high concentrations of ethanol (20%–50%) to create alcohosomes can increase the toughness of the vesicles and enhance permeation [18]. Ethosomes, compared to liposomes, improve the elasticity of the lipid bilayer and increase penetration into the deeper layers of the skin [35]. Limsuwan et al. [54] prepared an ethosomal formulation containing resorcinol and demonstrated that after application to porcine skin, the accumulation of resorcinol was 7.4 times higher than when using liposomes. Ethosomes also showed higher tyrosinase inhibition activity and decreased melanin content compared to liposomes.

Recently, a novel type of liposome called ginsenoside liposome has emerged as a carrier structure for active ingredients. Ginsenosides are the active components of ginseng with anti‐tumor properties [62]. These ginsenosides have a steroidal structure similar to cholesterol [63]. Ginsenosides have the ability to interact with phospholipids, enhancing the physical and chemical properties of the phospholipid bilayer similar to cholesterol [64, 65]. Studies have also shown that ginsenosides can serve as a substitute for cholesterol in liposomes [66]. Experimental evidence [67, 68] has demonstrated that the use of ginsenosides Rh2 and Rg2 instead of cholesterol in liposomes enhances their blood circulation and tumor‐targeting ability. Although direct data on the transdermal properties of ginsenoside liposomes have not been reported, it has the possibility of dermal absorption and also has good prospects for development and application in principle.

#### 4.2.2 Invasomes

Invasomes are flexible liposomes composed of phospholipids, ethanol, and a terpene molecule or mixture, similar to traditional liposomes [42, 69]. However, invasomes have stronger skin penetration compared to conventional liposomes [70]. Ethanol increases the fluidity of lipids in the skin's lipid bilayer and reduces the hardness of invasomes, making them more flexible and enhancing their permeability [71]. In addition to ethosomes, invasomes contain terpenoids, which can enhance permeability by disrupting the lipid bilayer structure of the stratum corneum [72]. Sanket et al. [69] compared the penetration rate of invasomes and conventional liposomes with azelaic acid, and the results showed that invasomes increased the penetration efficiency by approximately 1.5‐fold, exhibiting superior efficacy in acne treatment.

#### 4.2.3 Nanoemulsions

Nanoemulsions, mainly consisting of oil phase, water phase, and emulsifiers [43], are thermodynamically and dynamically stable nanoscale emulsions [44]. The droplet size of nanoemulsions is extremely small (20 to 400 nm) [73], and they are solid spheres with amorphous surfaces, lipophilic, and negatively charged [74]. Compared to conventional emulsions, nanoemulsions have more uniform droplet sizes, smaller particle sizes, and lower viscosity, which improve the stability and bioavailability of actives [75, 76]. Nanoemulsions have unique properties that can enhance dermal active delivery by altering the permeability of the stratum corneum, allowing actives to penetrate deeper into the skin [73]. This technology can also be used to address key challenges faced by essential oils [77] such as argan oil [78, 79] and rosemary oil [80, 81], which possess anti‐aging and antioxidant properties [78, 79]. Although essential oils can enhance their own permeability by altering the structure of the stratum corneum [82, 83], their inherent volatility and poor water solubility remain significant obstacles. Formulating essential oils into nanoemulsions ensures that oils like argan oil can be precisely delivered to the skin while maintaining their efficacy [84]. This protective and delivery‐enhancing effect applies not only to essential oils but also to other lipophilic bioactive substances. Research has shown that preparing CoQ10 as a nanoemulsion can enhance its solubility and permeability, with a release rate of up to 47.21% within 24 h. When applied to wrinkled skin, it reduces wrinkles, smoothes the skin surface, and reduces keratinization and hyperkeratinization in the epidermis [85].

#### 4.2.4 Cubosomes

Cubosomes refer to closed lipid bilayer structures with both bicontinuous water channels and lipid channels, spontaneously formed by dispersing amphiphilic lipid molecules in water or other polar solvents [45, 46]. The mechanism of cubosomes' enhancement of skin permeability is similar to that of liposomes and involves increasing the fluidity of the stratum corneum [46, 86]. This allows the actives contained within cubosomes to easily penetrate the mucous membranes and the epidermis of the skin [87], thereby promoting the effective encapsulation and sustained release of active therapeutic substances [88], and improving active bioavailability. In the study by Akhlagi et al. [89], researchers prepared plant triterpenoid cubes (Palpepcubes) containing two types of palmitoyl peptides, with the primary objective of slowing down the aging process and improving skin appearance. In vitro release studies showed that palmitoyl peptides are released continuously from Palpepcubes, which helps to stimulate collagen production and regulate cellular activity. Additionally, after being formulated into cubes, the storage stability of palmitoyl peptides was significantly improved. Sherif et al. [90] prepared cubosomes using lovastatin as a carrier for exhibiting photodamaged skin treatment efficacy. In the placebo‐controlled study, cubosomes‐treated samples showed a mean increase of 8.84% ± 4.93% in the combined thickness of the epidermis and dermis, compared to a 2.95% ± 2.14% increase in the placebo group. In tests conducted on volunteers, most participants experienced a reduction in facial fine lines, with almost complete disappearance of fine lines around the eye and upper lip areas. Therefore, the cubosomes show promise in the field of skincare active ingredient delivery.

#### 4.2.5 Dendrimers

Dendrimers are nanosized radially symmetric molecules with well‐defined, uniform, and monodisperse structures, consisting of a central core, internal branching structures (dendrites), and functional surface groups [47]. Dendrimers can enhance active solubility and also act as skin penetration enhancers [91, 92, 93]. They have the ability to accommodate various types of cargo, but are commonly used for the delivery of nucleic acids and small molecules [94, 95]. Zhao et al. [96] developed a self‐assembled dendritic conjugate system to achieve dermal delivery of isotretinoin, which enhanced the skin retention of isotretinoin, exhibiting greater therapeutic efficacy than free isotretinoin in a psoriasis model.

#### 4.2.6 Solid Lipid Nanoparticles

Solid Lipid Nanoparticles (SLNs) are nano or sub‐micron‐sized (average diameter of 50–1000 nm) colloidal carriers composed of solid lipids dispersed in an aqueous phase [48, 49], typically spherical in shape. SLNs are mainly composed of lipids and emulsifiers [3]. Depending on their composition, they can be classified as emulsions, where solid lipids serve as the oil phase. Both SLNs and liposomes are lipid‐based nano‐formulations that can serve as carriers for active substances [97]. However, compared to traditional liposomes, SLNs offer higher stability for the encapsulated active ingredients due to their rigid core lipid matrix [98, 99]. SLNs can be used in cosmetics as carriers for sunscreen, anti‐acne, anti‐aging agents, and fragrances [100, 101]. Chen et al. [102] demonstrated that encapsulating the antioxidant resveratrol in SLNs against UV‐induced degradation: a Phospholipon‐based formulation retained 92% and 83% of resveratrol at 4 and 6 h, respectively, whereas only approximately 28% remained in the free resveratrol solution after 6 h. Notably, the SLNs formulation also enhanced resveratrol's permeation through the stratum corneum, with an active release assay showing over 70% of resveratrol sustainably released within 24 h. Suter et al. [103] demonstrated that heptapeptide‐loaded SLNs significantly reduce skin DNA damage by 20% compared to traditional oil‐in‐water (o/w) formulations, thereby achieving anti‐aging effects in cosmetic applications.

#### 4.2.7 Polymersomes

Polymersomes are artificial vesicles made from amphiphilic block copolymers [50], which can encapsulate hydrophilic molecules in the aqueous core and hydrophobic molecules within the membrane [51, 104]. The hydrophilic segments improve biocompatibility, while the hydrophobic segments enhance the solubility and stability of hydrophobic actives [105]. They are similar to liposomes but offer improved stability, active retention efficiency, and relative ease of preparation [51, 106]. Recent studies have encapsulated essential oils in triblock copolymer shells to demonstrate effective control of head lice [107].

#### 4.2.8 Niosomes

The niosome is a nanoscale vesicle formed in water by a non‐ionic surfactant, cholesterol, or other amphiphilic molecule [52, 53], often single or multilayered, capable of encapsulating both hydrophilic and hydrophobic actives [108]. Their formation materials, non‐ionic surfactants, are superior to lipids in terms of physical and chemical stability, making them more stable than liposomes [108]. Additionally, they are cost‐effective alternatives to liposomes [109, 110] and can significantly improve the dermal absorption of active actives. For example, niosomes can improve the solubility and photostability of quercetin. They also have the advantages of sustained release and improved skin permeability. Skin retention was improved by 2.95‐fold compared to quercetin solution [111].

### 4.3 Ultrasound

Ultrasound can be defined as the application of ultrasound waves with frequencies ranging from 20 kHz to 16 MHz to disrupt the skin barrier for active delivery, with sufficient intensity to reduce skin resistance [20, 112], It can control the depth of penetration of active ingredients by changing the frequency of ultrasound, etc. [2], with non‐invasive, small damage to the skin and other characteristics [113]. Ultrasound can enhance skin permeability through mechanisms such as thermal effects and cavitation, enabling the effective delivery of various types and categories of actives [19], including hydrophilic actives and high‐molecular‐weight actives [114]. Cavitation is considered to be the primary mechanism in the sonophoresis process, disrupting the bilayer lipid structure of the stratum corneum and increasing active absorption [115, 116]. Coniti et al. [117] summarized data from the Global Aesthetic Improvement Scale (GAIS) to systematically evaluate the efficacy of microfocused ultrasound for facial skin tightening. The subjective investigator‐assessed rating scores (*n* = 337) collected on day 90 revealed that 92% of patients showed improvement in skin tightening and reduction of wrinkles, sustained for up to 1 year. YUE et al. [118] found that ultrasound‐assisted penetration technology can maximize the retention ratio of Rutin in the dermis layer. Compared with the non‐ultrasound method, the values are 1.8 times (using pig skin) and 2.63 times (using hairless mouse skin), respectively. In an inflammatory mouse model, skin barrier function was completely restored by day 4, significantly improving the dermal absorption efficiency of Rutin.

### 4.4 Invasive Techniques

#### 4.4.1 Microneedles

**FIGURE 3:**
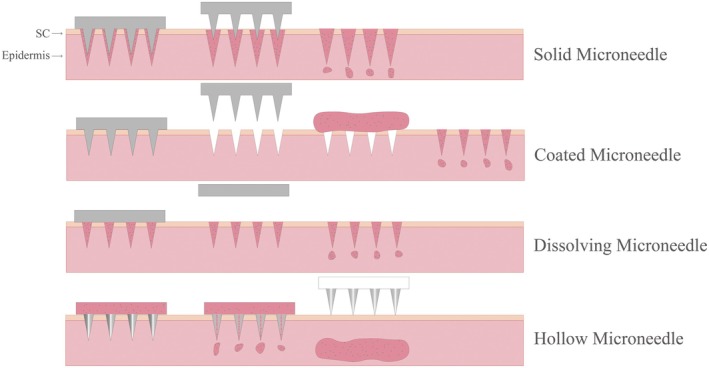
The active delivery mechanism of solid microneedle, coated microneedle, dissolving microneedle, and hollow microneedle.

Microneedles are arrays of micrometer‐sized needles of different materials [119, 120], which are used to administer actives by puncturing the skin and are generally as short as 25 μm in length and up to 1500 μm in length [121, 122], making them less invasive. The material of the needles can be silicon [123], metal [124], polymers [125, 126] and glass [127], among others. These needles are arranged on a small patch and are considered to be a hybrid of both hypodermic needles and transdermal patches [120]. The use of microneedles produces transient holes across the SC, forming tiny holes through which the channel partially diffuses into deeper layers of the skin [128, 129]. There are many types of microneedles, including solid microneedles, coated microneedles, dissolving microneedles, hollow microneedles, etc. [130, 131, 132]. As illustrated in Figure 3, in vitro experiments using the fluorescent dye calcein as a model drug showed that microneedle treatment significantly increased skin permeability: after inserting the microneedles for 10 s and removing them, calcein permeability increased by nearly 10 000 times; after inserting them for 1 h and removing them, permeability increased by approximately 25 000 times. This enhanced effect persists for at least 5 h in vitro and does not damage surrounding skin [133]. It also has been shown that microneedling can enhance the delivery of medicated peptides such as the anti‐wrinkle peptide rigin in the skin [134]. Due to the short length of the microneedle, it does not come into contact with the nerve fibers and blood vessels that touch the dermis [121, 135]. Human volunteer tests showed that inserting microneedles into the forearm or hand did not cause pain, only a slight feeling of pressure (similar to tape sticking to the skin), and skin resistance decreased 50‐fold after insertion. No adverse reactions such as redness or swelling were observed after the procedure, verifying its safety potential for cosmetic applications [133]. Compared to other dermal active delivery methods, microneedles are able to deliver larger sized active molecules without irritating the skin's nerve endings [136], thereby minimizing or completely avoiding the pain experienced by patients [128, 137].

There is also a type of soluble microneedle currently available [122]. The needle body is made from 100% active ingredients, which can better improve the bioavailability of the active ingredients. The cosmetic industry also uses skin rollers or microneedle devices to effectively treat scars, skin laxity, and wrinkles [138, 139]. The microneedle market is expected to continue growing at a compound annual growth rate of 7.1% until 2027 [140]. However, microneedles have a very small and fine needle size, and microneedle tip breakage may occur in the skin, which can be solved by changing the preparation material [120].

Some studies have also tried to use microneedles in combination with nanotechnology, such as Cubosomes [141, 142], liposomes [134, 143], polymersomes [139], and ultrasound [144]. Chen et al. [143] designed a novel hyaluronic acid (HA) containing curcumin polymersomes composite microneedle, which could be rapidly dissolved in the skin and delivered 74.7% of active loading to the skin within 6 h. It showed remarkable active penetration performance within a short period of time and has good prospects for application development. Petchsangsai [144] found that by combining the three techniques of microneedle, electroporation, and ultrasound, the macromolecular substance compound fluorescein isothiocyanate‐Dextran showed a good penetration and absorption effect with total skin accumulation greater than that observed using a single method or a combination of the two.

#### 4.4.2 Electroporation

Electroporation, also known as Electroacupuncture, is a phenomenon in which certain dielectric membranes, such as lipid bilayers, experience reversible electrical breakdown [145]. Electroporation involves the release of high‐voltage electric fields (generally ranging 50–300 V) in the form of pulses, which act on the lipid bilayer structure in the skin, creating new pores that facilitate the dermal delivery of active molecules [146, 147]. The efficiency of electroporation active delivery depends on variables such as applied voltage, duration, rate, and number of pulses [148]. Compared to microneedles, electroporation is more suitable for delivering small molecular substances, while microneedles are better suited for delivering large molecular actives [120]. Recently, Zaiyu Bioscience in Beijing has developed a nano‐electroporation beauty device that loads cell nutrients into nano‐electroporation probes, allowing for precise and safe skincare by delivering the nutrients to the epidermis and dermis layers of the skin. Marra et al. [149] used an electrotreatment device to deliver sodium ascorbyl phosphate (NAP) to pig ear skin. After 20 min of electrostimulation, samples were immediately collected. The penetration efficiency of NAP was 7.2 times higher than that of the normal administration control group (passive diffusion). When electrostimulation was followed by 60 min of normal administration, the penetration efficiency was 14.9 times higher than that of 80 min of control administration, demonstrating the superiority of the electrostimulation device in enhancing penetration efficacy.

However, electroporation technology cannot provide quantitative active delivery, and high‐field strength may lead to cell death [150] and potential damage to labile actives (such as protein‐based actives) [151].

Electroporation can also be combined with CPE [152], ultrasound [145], and other methods to improve active penetration. Sodium dodecyl sulfate (SDS) is a common chemical permeation enhancer [28]. Murthy et al. [153] found that SDS increased dermal absorption efficiency during electroporation by promoting barrier disruption during pulse application and extending the lifespan of electric pores.

#### 4.4.3 Needle‐Free Injector

Needle‐free injector (NFI) is a method of delivering actives without the use of needles. It involves using high‐velocity jets to puncture the skin and deliver molecules into the subcutaneous or intramuscular regions [154]. This method can avoid needle phobia and potential injuries caused by needle breakage within the body, and compared to traditional subcutaneous injections, NFI causes less pain [155, 156]. Needle‐free injector has a wide range of applications in treating skin diseases, such as alopecia, hyperhidrosis, psoriasis, and keloids [154, 157]. Bekkers et al. evaluated the efficacy, tolerability, and patient satisfaction of NFI‐assisted bleomycin therapy for severe keloids. Following NFI‐assisted bleomycin therapy, the estimated average keloid volume decreased significantly by 20%, and NFI treatment was preferred over previous needle injections in 85% of patients [158]. Currently, they are also being developed for use in the skincare industry [55, 159]. Research has shown that using jet injection of cross‐linked hyaluronic acid can effectively improve dermal moisture loss, skin condition, pores, and wrinkles [160].

## 5 Conclusion and Perspectives

As consumers deepen their understanding of skin aesthetics, their demands have expanded beyond the ingredients themselves to encompass the dermal absorption efficiency and targeted efficacy of active ingredients. The skin, serving as the primary site for cosmetic active ingredients, presents its stratum corneum's “brick‐and‐mortar structure” as the main barrier to dermal penetration. Large molecules exceeding 500 Da in molecular weight—such as peptides and proteins—struggle to penetrate independently [24]. The core characteristic of cosmetic dermal absorption lies in delivering active ingredients to specific skin layers without entering systemic circulation to produce whole‐body effects [161]. This principle defines the critical boundary for permeation‐enhancing technologies in cosmetics: any method that prevents systemic absorption while promoting precise delivery to target sites can be effectively applied.

From a technological application and practical efficacy perspective, mainstream dermal enhancement techniques have achieved widespread implementation in both cosmetic and medical aesthetic fields, demonstrating significant value. In cosmetics, chemical permeation enhancers and nanotechnology represent core application directions. Chemical enhancers facilitate penetration by increasing lipid fluidity in the stratum corneum and enhancing the thermodynamic activity of ingredients [25, 26, 27]. While concentration balancing is required to avoid skin irritation, rational formulation combinations can harmonize safety and efficacy. Nanotechnology leverages the unique structure of nanoscale carriers (such as liposomes and nanoemulsions) to enhance ingredient stability and solubility while increasing stratum corneum permeability for deep delivery [59, 73]. This significantly improves bioavailability while maintaining product aesthetics. Global cosmetics giants like L'Oréal Paris, Estée Lauder, and Lancôme have maturely integrated these technologies into product manufacturing. Examples include L'Oréal's Plentitude Revitalift Anti‐Wrinkle Firming Eye Cream and Lancôme's Hydra Flash Bronzer Day Moisturizer, which incorporate vitamin E in nano‐capsules. Both leverage nanotechnology to facilitate skin absorption of anti‐wrinkle and moisturizing benefits, meeting consumer demand for high‐performance cosmetics [162].

In medical aesthetics, physical dermal delivery technologies like ultrasound and microneedling have become pivotal solutions for overcoming large‐molecule ingredient delivery challenges and achieving precise cosmetic outcomes. Ultrasound technology alters the stratum corneum structure through mechanisms like cavitation, enabling targeted action on deeper skin layers. It is currently applied for facial and shoulder lifting and tightening treatments, effectively reducing wrinkles with lasting results [163]. Microneedling technology creates transient channels in the epidermis, bypassing the stratum corneum barrier to efficiently deliver active ingredients like anti‐aging compounds directly to target areas such as the superficial dermis. This plays a vital role in anti‐aging treatments [164]. Its minimally invasive nature and rapid recovery time have earned it widespread recognition in the medical aesthetics field.

It should be noted that while existing technologies have achieved certain breakthroughs, there remains room for optimization. Further refinement based on different skin types and aesthetic needs is necessary to achieve more precise efficacy delivery while reducing the risk of adverse reactions. For example, these approaches alter the skin's structure to some extent, affecting its permeability and potentially compromising its functions. Frequent interventions to facilitate the entry of external substances into the skin may disrupt the skin's equilibrium and lead to adverse consequences. Furthermore, although the aforementioned dermal delivery technologies have made significant advancements in the field of skincare, there are still limitations. For instance, some nanocarriers may pose toxicity and safety issues [34, 165]. Due to their small size, they can induce the production of reactive oxygen species and lead to DNA damage, inflammation, and other adverse reactions [166]. Therefore, future development requires not only continuous refinement of the technology itself but also a deeper understanding of skin structure and permeation mechanisms. This should drive proactive exploration of synergistic strategies for multiple dermal delivery technologies. Simultaneously, by optimizing formulation systems, strengthening quality research, and conducting rigorous clinical validation, a critical balance must be achieved between enhancing permeation efficiency and ensuring long‐term safety.

## Ethics Statement

The authors have nothing to report.

## Conflicts of Interest

The authors declare no conflicts of interest.

## Data Availability

Data sharing not applicable to this article as no datasets were generated or analysed during the current study.
